# Insight into the roles of lactylation in macrophages: functions and clinical implications

**DOI:** 10.1042/CS20242737

**Published:** 2025-01-13

**Authors:** Min Shu, Dingci Lu, Ziyi Zhu, Fei Yang, Zhaowu Ma

**Affiliations:** 1 School of Basic Medicine, Health Science Center, Yangtze University, Nanhuan Road 1, Jingzhou, Hubei 434023, China

**Keywords:** human diseases, lactylation, macrophages, post-translational modification, regulatory proteins

## Abstract

Lactylation, a post-translational modification, has been linked to gene transcription regulation through epigenetic modulation in various pathophysiological processes. The lactylation regulatory proteins, known as writers, erasers, and readers, govern their dynamics by adding, removing, and recognizing lactyl groups on proteins. Macrophages, as cells of the immune system, maintain homeostasis, responding dynamically to diverse internal and external stimuli. Emerging researches unveil that lactylation, through inducing macrophage activation and polarization, affects their functionality in pathological conditions such as inflammation, tumor microenvironment, and fibrosis. Evidence progressively indicates that lactate-driven alterations in lactylation levels within macrophages can influence the pathogenesis of numerous diseases. This review aims to systematically summarize the research progress of lactylation in macrophages, explore its functions and mechanisms by which lactylation contributes to the pathology of different disease phenotypes, and propose future research directions along with potential diagnostic and therapeutic strategies.

## Introduction

Lactylation is a form of post-translational modifications (PTMs), which also encompass glycosylation, acetylation, methylation, ubiquitination, and phosphorylation, all of which are vital for the modulation of protein function [1,2]. Protein lactylation significantly influences various biological processes such as tumor growth, neural activation, immune responses, and inflammatory responses by regulating gene transcription and protein function, making it a new focus for studying human diseases [3-5]. This modification has emerged as a focal point for understanding cellular metabolic reprogramming, epigenetic regulation, and potential implications in human diseases [6-8]. Increasing evidence indicates that lactylation can regulate transcription by histone modification and modulation of non-histone protein function. Among these, histone lysine lactylation (Kla) is the most common. Lactate serves as a precursor that can promote histone lactylation and influence the levels of histone Kla. Research has established that L-lactate is a recognized precursor in the histone Kla modification process, which is likely facilitated by the catalytic activity of lactyl coenzyme A [9-11]. Notably, L-lactate is the predominant form found in humans, and the process of histone lactylation predominantly encompasses lysine L-lactylation [12-14]. In recent times, lactylation has emerged as a significant focus of interest within the realm of PTMs of proteins. This modification entails the incorporation of lactate onto lysine residues found in histones and various other proteins, subsequently influencing gene expression and cellular activities [15]. Lactate, traditionally viewed as a mere byproduct of anaerobic glycolysis, has now been recognized for its multifaceted roles in cellular metabolism and signaling [16]. Lactate promotes tissue repair gene expression, such as arginase-1 (Arg1), through histone lactylation and interleukin-6 (IL-6) signaling [17]. Among the various metabolic byproducts, lactate has emerged as a pivotal player, influencing a myriad of biological processes through its role in lactylation. It functions as an essential energy source and a signaling entity, coordinating numerous physiological and pathological mechanisms. Recent research has highlighted the significant role of lactate-driven lactylation in DNA damage repair [18]. Lactylation contributes to genomic stability and chemotherapy resistance, and inhibiting lactate production and promoting lactylation of certain genes may be a promising strategy for cancer therapy [19-21].

Macrophages, as players in the immune system, are crucial immune cells involved in inflammatory and immune responses within the immune system. They help recognize and eliminate pathogens and also regulate immune responses through phagocytosis, antigen presentation, and cytokine secretion [22-25]. Furthermore, the polarization state of macrophages significantly influences their functional outcomes: M1 macrophages primarily mediate pro-inflammatory responses, while M2 macrophages facilitate anti-inflammatory processes and tissue repair [26-29]. Moreover, macrophages exhibit dual properties within the tumor microenvironment, displaying the capacity to both inhibit tumor progression and promote tumor growth [30,31].

Emerging research into cellular metabolism has revealed that lactate, beyond being a metabolic byproduct, acts as a signaling molecule that participates in the regulation of macrophages [6]. Studies have found that lactylation can affect the activation state of macrophages, thereby influencing their functions in inflammation and tumor microenvironments [32]. Similarly, lactylation of specific proteins in macrophages has been linked to the suppression of inflammatory responses, highlighting its potential as a therapeutic target for inflammatory diseases [33]. In the context of metabolic diseases, lactylation has been demonstrated to be essential in the modulation of lipid metabolism and sensitivity to insulin [34]. Furthermore, lactylation is vital for modulating the immune system, affecting the functionality of immune cells like T cells [35,36]. Lactylation is linked to immune cell regulation in autoimmune diseases, such as Crohn’s disease, by influencing immune cell infiltration and inflammation [37]. In addition, the role of lactylation in the tumor microenvironment has also received considerable attention. Research has shown that lactylation regulates the polarization state of tumor-associated macrophages (TAMs), promoting tumor growth and metastasis [38].

Current reviews have focused on the role of lactylation in respiratory diseases, cancer, and metabolic disorders [39-41]. Despite the potential importance of lactylation in macrophage function, there remains a significant gap in knowledge regarding the role of the macrophage-lactylation link in various human diseases. This review systematically elucidates the distinct functional roles of lactylation in macrophage diversity and phenotypic variability in a series of human diseases. Furthermore, potential clinical applications of lactylation as promising diagnostic biomarkers or therapeutic targets in human diseases have been discussed, striving to offer novel perspectives and pathways for research in the fields of lactylation and macrophages.

## Lactylation modifications

PTMs are vital for regulating protein function, stability, and interactions within the cell [42-44]. Among these PTMs, lactylation has gained attention due to its regulation of gene transcription and protein function [45,46]. Lactylation has been shown to affect the expression of oncogenes and tumor suppressor genes, and it can also modify proteins to influence various cellular processes [47,48]. Lactylation is primarily driven by the accumulation of lactate, which has traditionally been viewed merely as a byproduct of glycolysis. However, it is now recognized as a signaling molecule that can modulate immune responses and facilitate metabolic reprogramming [49,50]. Lactylation under high lactate conditions, influenced by pH, temperature, and cofactors, enhances histone modification and gene expression in tumors [51]. Its specificity relies on lysine context and protein structure, affecting protein function and signaling in cancer and metabolic disorders, guiding targeted therapy research [52-54].

**Figure 1:**
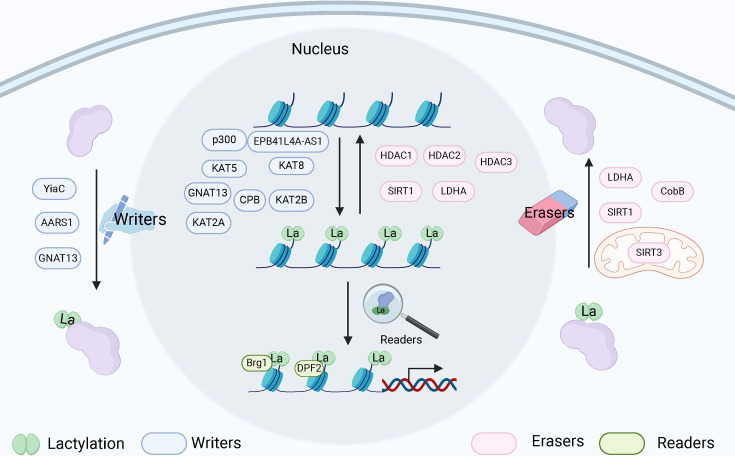
Functions and roles of writers, erasers, and readers in lactylation. The writers, erasers, and readers modify proteins by adding, removing, or interpreting lactylation groups (created with BioRender.com).

Understanding the regulatory proteins that mediate protein lactylation modifications is crucial for elucidating the biological significance of this PTM. The formation of lactylation relies primarily on the reaction of lactic acid with protein lysine residues through the catalytic action of specific enzymes. Lactylation is mediated by specific enzymes that facilitate or repress the transfer of lactate moieties to lysine residues on proteins, a process that is similar to acetylation. The primary enzymes implicated in this modification are lactate acyltransferase (lactyltransferases) and de-lactylases (de-lactylases), which have been identified in various cellular contexts, particularly in cancer cells [9,54]. The regulation of chromatin architecture via PTMs has surfaced as a pivotal factor in transcriptional reactions across various cell types [55]. Similarly, the regulatory proteins of the writer, eraser, and reader apparatus, which are responsible for adding, removing, or recognizing these lactylation modifications, have become integral players in our understanding of physiological responses in diverse cellular contexts (Figure 1) [56,57]. First, lactylation “writers” are enzymes or proteins that catalyze the lactylation reaction, which can facilitate the interaction between lactate and specific functional groups within the target molecule, resulting in modification by incorporating lactyl groups. Most recently, researchers have identified the acetyltransferases AARS1, CPB, EPB41L4A-AS1, GNAT 13, KAT2A, KAT2B, KAT5, KAT8, p300, TIP60, and YiaC as potential catalyzed lactylation [11,21,58-67]. Second, lactylation “eraser” refers to an enzyme or protein possessing the ability to remove or erase lactylation groups through hydrolysis, thus restoring the target molecule to its original state. For example, lactate dehydrogenase A (LDHA), sirtuin-1,3 (SIRT1,3), histone deacetylases (HDACs 1–3), and cobB (NAD-dependent protein deacylase) demonstrate delactylase activity [10,20,60,68-72]. Third, lactylation “reader” refers to a protein or domain capable of recognizing and interacting with lactylation groups. Recent reports have identified Brg1 as the histone lactylation reader that interacts with H3K18la to facilitate the reprogramming process [73]. A recent novel finding reveals that DPF2 is a reader of H3K14la, which links histone lactylation to gene transcription and cell survival [74]. The lactylation cycle, governed by the actions of writer and eraser enzymes, represents a crucial regulatory mechanism in cellular metabolism and gene expression. The intricate balance between lactylation and delactylation is essential for maintaining cellular homeostasis and responding to metabolic changes.

In summary, the writers, erasers, and readers modify proteins by adding, removing, or interpreting lactylation groups (Figure 1 and Table 1), impacting key cellular processes like stability, localization, enzyme and transcriptional activity, and interactions, thus regulating physiological and pathological processes. Understanding lactylation-regulatory enzymes is crucial for grasping lactylation’s role in human diseases, particularly in metabolic disorders and cancers.

**Table 1:** Writer, reader, and eraser proteins of lactylation modifications.

Regulatory proteins	Full name/alternative name	Function	Cellular localization	Protein lactylation sites	References
**Writers of lactylation**					
AARS1	Alanyl-tRNA synthetase 1	Lactyltransferase	Cytoplasm	YAP K90 and TEAD1 K108,	[61]
CPB	CREB-binding protein C and histone acetyltransferase	Acetyltransferase	Nucleus	HMGB1, MRE11 K673	[21,75]
EPB41L4A-AS1	Erythrocyte membrane protein band 41-Like 4A Antisense RNA 1	Acetyltransferase	Nucleus	Undefined	[76]
GNAT 13	Gcn5-related N-acetyltransferase 13	Lactyltransferase	Membrane and cytoplasm	RpoA K173	[62]
KAT2A	GCN5	Acetyltransferase	Nucleus	H3K18, H3K9	[59,63]
KAT2B	PCAF, GCN5L2	Acetyltransferase	Nucleus	TBX5 K339	[64,65]
KAT5	Tip60	Acetyltransferase	Nucleus	Undefined	[58]
KAT8	Lysine acetyltransferase 8, MYST1	Acetyltransferase	Nucleus	LTBP1 K752, eEF1A2 K408	[66,67]
p300	KAT3A, KAT3B	Catalyze the transfer of the lactyl group from lactyl-CoA to histones	Nucleus	HMGB1, H3K27, H3K18,	[11,63,75]
TIP60	Acetyltransferase of MYST family	Lactyltransferase	Nucleus	NBS1 K388	[20]
YiaC	Peptidyl-lysine N-acetyltransferase YiaC	Peptidyl-lysine N-acetyltransferase YiaC	Cytoplasm (bacterial cells)	Undefined	[60]
**Erasers of lactylation**					
CobB	NAD-dependent protein deacylase	NAD-dependent protein deacylase	Cytoplasm (bacterial cells)	PykF K382la	[60,68]
HDAC1	Histone deacetylase 1	Removes acetyl groups from histones	Nucleus	H3K18, H4K5	[10]
HDAC2	Histone deacetylase 2	Removing acetyl groups from histones	Nucleus	H3K18, H4K5	[10,69]
HDAC3	Histone deacetylase 3	Class I histone deacetylases	Nucleus	H3K18, H4K5, NBS1 K388	[10,20]
LDHA	Lactate dehydrogenase A	The glycolytic enzyme that catalyzes the conversion of pyruvate to lactate	Cytoplasm (primarily)	TTK protein kinase (TTK)	[20,69]
SIRT1	Sirtuin-1	NAD-dependent protein deacetylase sirtuin-1	Nucleus and cytoplasm	α-MHC	[70,71]
SIRT3	Sirtuin-3	NAD-dependent protein deacetylase	Mitochondria	Undefined	[72]
**Readers of lactylation**					
Brg1	Brahma-related gene 1	Core component of the SWI/SNF chromatin remodeling complex	Nucleus	H3K18	[73]
DPF2	Decreased expression in fat 2	Modifying chromatin structure and control cell proliferation and differentiation	Nucleus	H3K14la	[74]

## Lactylation influences the functions of macrophages

Lactylation has emerged as a significant regulatory mechanism in macrophages, influencing their function and phenotype. This modification involves the addition of lactate-derived lactyl groups to lysine residues on histones and non-histone proteins, thereby impacting gene expression and cellular processes. The role of lactylation in macrophages is multifaceted, affecting various aspects of their biology, including metabolism, inflammation, and immune response [77]. One of the primary mechanisms by which lactylation influences macrophages is through the regulation of gene transcription, protein PTM, and cell survival.

Lactylation of histone and non-histone significantly affects macrophage inflammation to change cellular metabolic states. Histone lactylation emerges as a critical regulator of reparative gene expression in monocytes following myocardial infarction (MI), facilitating both anti-inflammatory and pro-angiogenic activities in macrophages. Elevated levels of histone lactylation, specifically H3K18 modification, are associated with the transcriptional up-regulation of essential reparative genes, including IL-10, Vegf-a, and Lrg1, ultimately contributing to enhanced cardiac performance following MI. This research highlights the significance of metabolic reprogramming within monocytes and emphasizes the function of IL-1β-dependent recruitment of GCN5 in the regulation of histone lactylation, thereby promoting a reparative environment during the early phases of cardiac injury [59]. Non-histone lactylation found that monocarboxylic acid transporter proteins 4 (MCT4) dependent lactate transport in exacerbating cardiac energy metabolism injury and inflammation associated with diabetic cardiomyopathy (DCM) in type 2 diabetes. The up-regulation of MCT4 facilitates excessive lactate efflux from cardiomyocytes, leading to oxidative stress and enhanced histone H4K12 lactylation in macrophages, which further contributes to inflammatory infiltration within the myocardial microenvironment. Importantly, inhibiting MCT4 mitigates oxidative damage and inflammatory responses, suggesting a novel therapeutic approach for preserving cardiac function in diabetic patients by targeting lactate-mediated pathways [78]. These findings highlight the bifunctional nature of lactylation, which can either facilitate or mitigate inflammation, contingent upon the specific context and targeted entities involved.

Lactylation serves as an epigenetic mark that can reprogram macrophage gene expression profiles. This modification is particularly significant in the context of chronic inflammatory diseases and cancer. For instance, in ocular melanoma, histone lactylation has been shown to drive oncogenesis by facilitating the expression levels of the m6A reader protein YTHDF2, which in turn promotes tumor growth and metastasis. This modification facilitates the expression of the m6A reader protein YTHDF2, which subsequently promotes the degradation of m6A-modified PER1 and TP53 mRNAs, thereby accelerating tumorigenesis. These findings not only establish a link between histone lactylation and RNA modifications but also identify novel therapeutic targets for the treatment of ocular melanoma [79].

Lactylation is intricately linked with metabolic pathways, particularly glycolysis. The relationship between lactate synthesis and lactylation highlights the metabolic alterations that take place in macrophages in response to inflammation and cancer. Histone lactylation, specifically H4K12 lactylation, is significantly elevated in microglia (the resident macrophages of the brain) from both Alzheimer’s disease (AD) patients and 5XFAD mice, promoting the transcription of glycolytic genes, the positive feedback loop involving glycolysis, H4K12la, and PKM2 intensifies the dysfunction of microglia, and disrupting this loop has been shown to improve both microglial dysfunction and amyloid-beta (Aβ) pathology. Importantly, disrupting this feedback loop through pharmacological inhibition of PKM2 or specific ablation in microglia enhances cognitive function and reduces Aβ pathology, highlighting lactylation as a potential therapeutic target in AD [80]. Moreover, increased lactate concentrations within the tissue microenvironment trigger both metabolic and epigenetic reprogramming of pro-inflammatory Th17 cells, leading to diminished IL-17A production and enhanced Foxp3 expression. Notably, lactate treatment resulted in increased histone H3K18 lactylation levels in Th17 cells, a modification previously associated with active chromatin in macrophages, underscoring lactate’s role in modulating T-cell function. High lactate concentrations can suppress Th17 pathogenicity and promote a shift toward regulatory T cell-like phenotypes, highlighting lactate’s dual role in inflammation and immune regulation [81]. Non-histone lactylation, in non-alcoholic fatty liver disease (NAFLD), mitochondrial pyruvate carrier 1 (MPC1) regulates fatty acid synthase (FASN) lactylation, which in turn affects lipid metabolism and inflammation in hepatocytes. In NAFLD, there exists a positive correlation between the levels of MPC1 and the accumulation of hepatic lipids, and the increased lactylation at the K673 site of FASN may represent a downstream mechanism contributing to this process [82]. Consequently, lactylation has the potential to influence the equilibrium between inflammatory damage and anti-inflammatory repair within tissues by altering the metabolic reprogramming mechanisms in macrophages.

Macrophage polarization is a dynamic process influenced by various metabolic and epigenetic factors, including lactylation. The differentiation of macrophages into pro-inflammatory M1 or anti-inflammatory M2 phenotypes plays a crucial role in the immune response and the maintenance of tissue homeostasis. Lactylation has been identified as a crucial modulator of this mechanism. For example, histone lactylation serves to amplify M2 gene expression, thereby promoting anti-inflammatory functions. The regulation of macrophage transformation from an inflammatory to a reparative state is orchestrated by B-cell adapter for PI3K (BCAP) through histone lactylation. In the absence of BCAP, macrophages exhibit a notable impairment in glycolysis and lactate production, which in turn diminishes histone lactylation and the expression of genes associated with tissue repair, ultimately obstructing the recovery process [83]. This shift is crucial for resolving inflammation and promoting tissue repair. For example, lactylation of histone H3 at lysine 18 (H3K18) has been implicated in the activation of genes involved in tissue repair and anti-inflammatory responses, thereby facilitating the resolution of inflammation [84]. Moreover, non-histone lactylation, the accumulation of lactate resulting from aerobic glycolysis in M1 macrophages induces lactylation modification of PKM2 at the K62 position. This modification activates PKM2, promoting its conversion into a tetrameric form while concurrently diminishing its dimerization and nuclear translocation. Consequently, this process suppresses the Warburg effect in macrophages, leads to a reduction in lactate production, and supports the transition of M1 macrophages toward the M2 repair phenotype [33]. Additionally, the tumor cell-derived lactate is a key driver of polarization in TAMs, operating independently of MPC-mediated metabolism. The genetic reduction in MPC effectively blocks lactate entry into macrophage mitochondria but does not impede IL-4/lactate-induced macrophage polarization, HIF-1α stabilization, or histone lactylation. Hence, the findings underscore that lactate itself, rather than its mitochondrial metabolites, is critical for promoting the polarization of TAMs, highlighting a novel perspective on lactate’s role in the tumor microenvironment [85]. These studies underscore the importance of lactylation in modulating macrophage polarization and its potential therapeutic implications. Therefore, lactylation represents a critical regulatory mechanism in macrophages, influencing their inflammatory responses, epigenetic reprogramming, metabolism, and polarization. Understanding the intricate details of lactylation and its impact on macrophage function could pave the way for innovative treatment approaches in treating inflammatory and metabolic diseases.

## The roles of macrophages lactylation in human diseases

**Figure 2:**
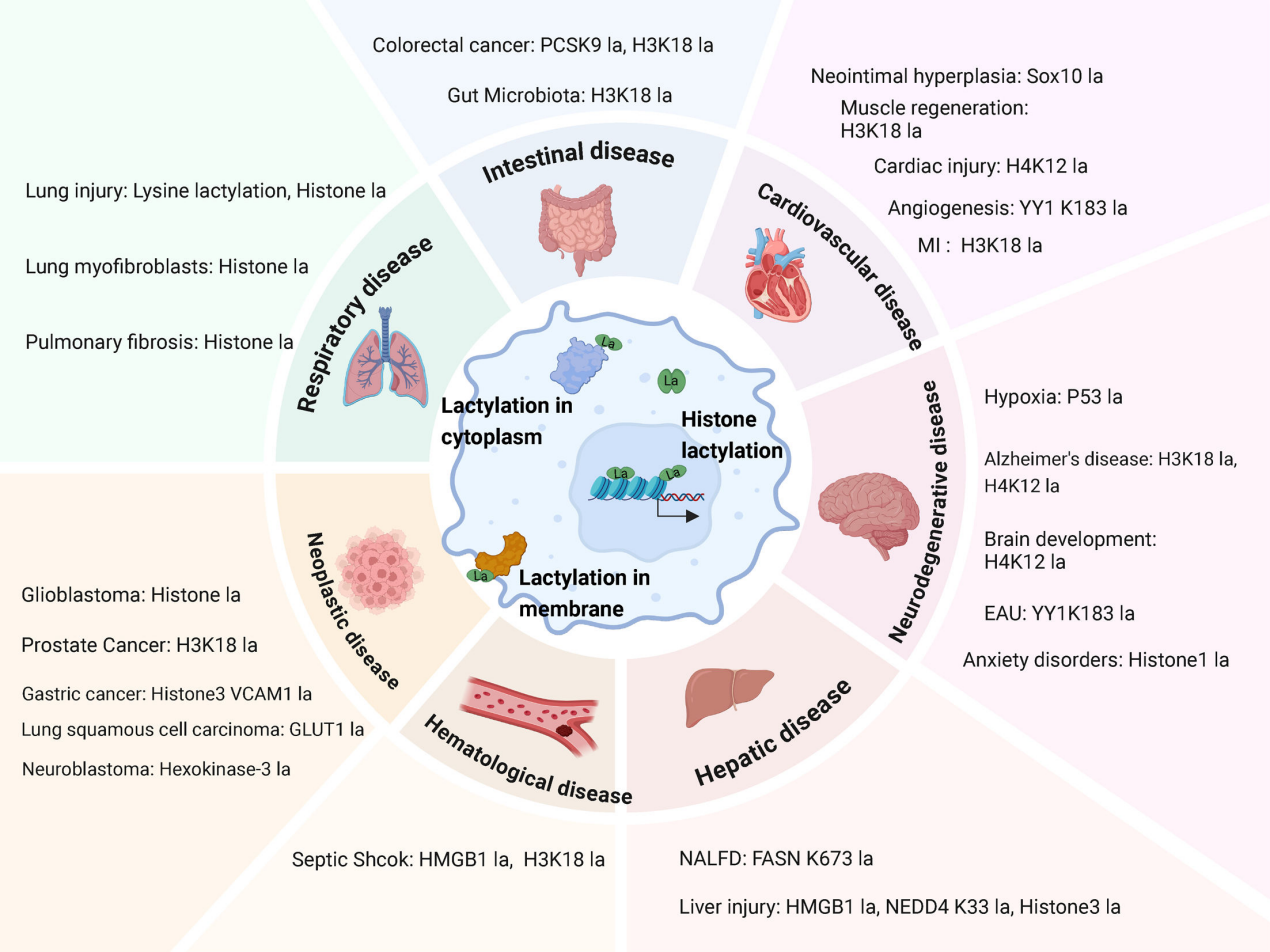
Macrophages of lactylation in relation to disease regulation. Lactylation of macrophages occurs at modification sites associated with various disease phenotypes, including respiratory disease, intestinal disease, cardiovascular diseases, neurodegenerative diseases, hepatic diseases, hematological diseases, and neoplastic diseases (created with BioRender.com).

Lactylation has become an essential modulator in numerous cellular functions, particularly in macrophages. This modification entails the incorporation of lactate onto lysine residues on proteins, influencing their function and stability. Therefore, lactylation represents a crucial PTM that influences their roles in various human diseases. In the following sections, we focus on the expanding landscape of macrophages lactylation in human diseases, including neoplastic disease, respiratory disease, cardiovascular diseases, central nervous system diseases, intestinal disease, hepatic disease, and hematological disease (Figure 2 and Table 2)

**Table 2:** Effects of macrophages by modifying lactylation in various diseases.

Protein targets	Lactylation sites	Gene expression	Enzyme activity	Disease/ outcome	Function	References
PKM2	K62	ARG1↑, iNOS↓	↑	Macrophage phenotype transition	Inhibits tetramer-to-dimer transition of PKM2	[33]
Histone 4	K12	Undefined	↑	Cardiac injury	Facilitating inflammatory infiltration	[78]
Histone 3	K18	Undefined	↑	Promote homeostasis	Induces homeostatic genes that are involved in wound healing	[11]
Histone 3	K18	IL-17A, FOXP3 ↑	Undefined	Reprogramming pro-inflammatory T cell phenotypes into regulatory T cells	High lactate concentrations suppress Th17 pathogenicity	[81]
Histone 3	VCAM1	CXCL1↑	↑	Gastric cancer	Facilitate human gastric cancer-derived mesenchymal stem cells and M2 macrophage recruitment and infiltration	[86]
Histone 3	K18	Undefined	Undefined	Prostate cancer	Suppressed histone lactylation within TAM, resulting in their anticancer phagocytic activation	[87]
Histone 3	K18	Undefined	Undefined	Prostate cancer	Durable ADT-independent tumor control in PTEN/p53-deficient AVPC	[88]
GLUT1 ↑	Undefined	SLC2A1 ↑	↑	Lung squamous cell carcinoma	Inhibition blocked macrophage polarization under hypoxia	[89]
Histone	Undefined	IL-10↑	Undefined	Glioblastoma	Promotes IL-10 expression required for suppression of T cell activity	[90]
HK3	Undefined	Undefined	Undefined	Neuroblastoma	HK3 not only directly regulates the malignant biological behaviors of tumor cells, such as proliferation, migration, and invasion, but also recruits and polarizes M2-like macrophages through the PI3K/AKT-CXCL14 axis in neuroblastoma	[91]
Lysine lactylation	Undefined	Undefined	Undefined	Lung injury	Global profiling of lysine lactylation in human lungs	[92]
Histone	Undefined	P300	Undefined	Lung myofibroblasts	Myofibroblast glycolysis	[93]
Histone	Undefined	Undefined	↓	Lung injury	Anti-inflammation	[94]
Histone	Undefined	Undefined	Undefined	Pulmonary fibrosis	Triggering glycolysis and subsequent modification of histone lactylation in macrophages	[95]
Sox10	Undefined	C3, CD74, and LYZ2↑	↑	Neointimal hyperplasia	Promoted pyroptosis	[96]
YY1	K183	FGF2↑	↑	Angiogenesis	Enhances FGF2 transcription and promotes angiogenesis	[97]
Histone 3	K18	ARG1, VEGF-a, and IL-10↑	↑	MI	Anti-inflammatory and pro-angiogenic	[59]
Histone 3	K18	Undefined	Undefined	Muscle regeneration	Muscle regeneration	[98]
Histone 3	K18	Rela (p65↑, NFκB1 (p50)↑)	Undefined	AD	Up-regulating senescence-associated secretory phenotype components IL-6 and IL-8	[99]
Histone 4	K12	PKM2	Undefined	AD	Exacerbating glucose metabolism disorders and microglial dysfunction in AD	[80]
Histone 3	Undefined	Undefined	Undefined	AD	Promoted the expression of a repair phenotype in mouse microglia	[100]
Histone 4	K12	Lrrc15	↑	Brain development	Microglia Bach1-deficient mice show anxiety-like behavior	[101]
YY1	K183	FGF2	↑	EAU	Boosting microglial activation and promoting their proliferation and migration abilities	[102]
P53	Undefined	iNOS, IL-6, IL-1β, TNFα↑		Hypoxic	Proinflammatory activation	[103]
PCSK9	Undefined	Undefined	Undefined	Colon cancer	M2 macrophage polarization	[104]
Histone 3	K18	MCT1	Undefined	Gut microbiota	Suppressing macrophage pyroptosis and modulating the intestinal microbiota	[105]
Histone 3	K18	RARγ↓️STAT3 ↑	Undefined	Colorectal tumorigenesis	Prohibit RARγ gene transcription, enhancing IL-6 levels and activation transcription 3 (STAT3) signaling	[106]
FASN	K673	MPC1↑	↑	NALFD	Hepatic lipid deposition	[82]
HMGB1	Undefined	Undefined	Undefined	Liver injury	Macrophage chemotaxis/activation	[107]
HMGB1	Undefined	SIRT1↑, GPR81↑	↑	Septic shock	Increases endothelium permeability	[75]
Histone 3	K18	Undefined	Undefined	Septic shock	Romotes the expression of Arg1 by lactate-H3K18la pathway to control macrophage to M2 polarization	[108]
Histone 3	K18	ARG1↑	Undefined	Septic shock	Mediate inflammatory cytokine expression and Arg1 overexpression and stimulate the anti-inflammatory function of macrophages in sepsis	[109]
NEDD4	K33	Undefined	Undefined	Liver injury	Restraining lactylation reduces non-canonical pyroptosis in macrophages and ameliorates liver injury	[32]
Histone 3	Undefined	LDHA↓	↓	Liver injury	Downregulating the level of LDHA in the treatment of liver injury	[34]

### Neoplastic disease

In neoplastic diseases, lactylation has been recognized as an essential modulator of TAMs and tumor progression. Lactate, produced by rapidly proliferating tumor cells, can induce histone lactylation in TAMs, thereby influencing their activity and promoting tumor growth. This modification has been linked to the regulation of genes involved in tumor progression and immune evasion. For example, the lactylation of H3K18 has been shown to enhance the expression of vascular cell adhesion molecule 1 (VCAM1), thereby facilitating the progression and metastasis of gastric cancer through the AKT-mTOR-CXCL1 signaling pathway. Specifically, VCAM1 elevates the expression of CXCL1 via the activation of the AKT-mTOR pathway, which subsequently promotes the recruitment and infiltration of human gastric cancer-derived mesenchymal stem cells and M2 macrophages. Targeting H3K18 lactylation and VCAM1 could be a potential therapeutic approach for gastric cancer [86].

In the tumor microenvironment, lactate accumulation due to the Warburg effect leads to extensive lactylation of histones and non-histone proteins in macrophages. This modification has been implicated in the polarization of TAMs toward a pro-tumorigenic phenotype, thereby facilitating tumor progression and metastasis. In the context of prostate cancer, the modulation of lactate levels alongside the inhibition of PD-1-mediated immunosuppression in macrophages plays a crucial role in regulating the proliferation of prostate cancer cells that are deficient in PTEN and p53. Inhibition of lactate production mitigates the suppression of TAM induced by histone lactylation and improves the phagocytic activity of TAM toward cancer cells [87]. Additionally, the reduction in lactate synthesis observed in PTEN/p53-deficient pancreatic cancer (PC) cells following treatment with PI3K inhibitors, MEK inhibitors, and PORCN inhibitors leads to a suppression of histone lactylation and promotes the phagocytic activity of TAM toward these PC cells. Inhibiting lactate production in tumor cells leads to enhanced phagocytosis by macrophages, providing a potential treatment strategy for aggressive-variant prostate cancer [88,110].

In addition, the increased expression of GLUT1 facilitated the polarization of macrophages toward the M2 phenotype. SLC2A1 has been identified as a potential predictive biomarker for both survival outcomes and the efficacy of immunotherapy in lung squamous cell carcinoma [89]. PERK-driven glucose metabolism promotes monocyte-derived macrophages immunosuppressive activity via histone lactylation in glioblastoma [90]. Moreover, the extensive dataset initially indicated that HK3 has the potential to influence glycolytic pathways, facilitate malignant biological activities, and serve as a prognostic marker for the aggressive progression of clear cell renal cell carcinoma. Furthermore, HK3 may enhance the presence of infiltrating monocytes and macrophages that exhibit specific surface markers, while also modulating critical molecular subgroups associated with immune checkpoint molecules on exhausted T cells. This action may consequently shape the microenvironment to favor active antitumor immune responses [91].

Investigations into the molecular mechanisms underlying lactylation’s role in facilitating tumor progression, along with the advancement of targeted antitumor pharmacological agents derived from these insights, remain ongoing. The exploration of therapeutic strategies aimed at the metabolic and signaling pathways associated with lactate production, as well as the transport and recognition of lactate between tumor cells and macrophages, represents a relatively emerging domain within the broader context of antitumor therapeutic research [111].

### Respiratory disease

Lactylation has been associated with a range of respiratory disorders, such as chronic obstructive pulmonary disease (COPD) and asthma. This modification holds importance in the inflammatory response and tissue repair mechanisms. For instance, in a study on necroptosis in pulmonary diseases, it was found that lactate-mediated lactylation of histones in macrophages can exacerbate inflammation by promoting the release of damage-associated molecular patterns [92].

This shift is critical in the pathogenesis of diseases like COPD, where chronic inflammation leads to tissue damage and impaired lung function. Furthermore, lactate has been shown to induce histone lactylation at the promoters of profibrotic genes within macrophages, aligning with the observed increase in this epigenetic modification in these cells found in fibrotic lung tissue. Underlying the key contribution of myofibroblast glycolysis to the pathogenesis of lung fibrosis [93]. Similarly, the study highlights the role of glycolysis and histone lactylation in macrophages in the pathogenesis of PM2.5-induced pulmonary fibrosis [95]. Moreover, the examination of infiltrating macrophages within the pulmonary tissue revealed that the Cur-RV treatment induced polarization of macrophages toward the M2 phenotype. Additionally, the observed reduction in histone lactylation may play a role in mediating their anti-inflammatory effects [94].

### Cardiovascular disease

Lactylation has emerged as an influence on various cellular processes, including those involved in cardiovascular diseases. Recent studies have highlighted the role of histone lactylation in macrophages, particularly in the context of MI. Histone lactylation has been shown to boost reparative gene activation post-MI, facilitating the timely activation of reparative signals crucial for the resolution of inflammation and the restoration of cardiac function. For instance, histone H3K18 lactylation in monocyte-macrophages early post-MI has been linked to the up-regulation of anti-inflammatory and pro-angiogenic genes, including Vegf-a, Lrg1, and IL-10. These genes play a vital role in cardiac repair processes and the enhancement of cardiac function [59].

Moreover, the dynamics of histone lactylation play a crucial role in the functionality of macrophages throughout the process of muscle regeneration. It has been established that histone lactylation plays a significant role at both promoter and enhancer regions. Furthermore, the genomic profile of H3K18 lactylation exhibits alterations between 2 and 4 days post-infection (dpi), which serve as predictors for subsequent changes in gene expression, rather than merely reflecting alterations in gene expression that occurred earlier [98]. Sox10 facilitates vascular inflammation through its role in the transdifferentiation of vascular smooth muscle cells (VSMCs) and the induction of pyroptosis during neointimal hyperplasia. An increase in Sox10 expression correlates with the accumulation of macrophage-like VSMCs and the occurrence of pyroptosis. Conversely, the down-regulation of Sox10 results in a reduction in vascular inflammation and neointimal hyperplasia [96].

Furthermore, macrophage lactylation has been associated with the modulation of immune responses during cardiovascular diseases. For example, the inhibition of macrophage MCT4, a lactate transporter, has been shown to activate reparative genes and protect against atherosclerosis by promoting histone H3K18 lactylation. This modification enhances the regulation of genes associated with tissue repair and inflammation resolution, highlighting the therapeutic potential of targeting lactylation pathways in cardiovascular diseases [112]. Yin Yang 1 (YY1), a nonhistone transcription factor, undergoes lactylation at lysine 183 (K183), a modification that is modulated by the p300 acetyltransferase. The hyperlactylation of YY1 significantly augments the transcription of FGF2, thereby facilitating angiogenesis. In the context of microglia, YY1 lactylation is crucial for retinal neovascularization, primarily through the up-regulation of FGF2 expression [97]. In addition, lactylation modifications influence macrophage functions, including lipid metabolism and inflammatory responses, thereby affecting plaque stability and progression in atherosclerosis [38].

Overall, the emerging evidence underscores the critical role of lactylation in modulating macrophage function and gene expression during cardiovascular diseases. By influencing key metabolic and inflammatory pathways, lactylation represents a promising target for therapeutic interventions aimed at improving cardiovascular health and mitigating disease progression.

### Neurodegenerative disease

In neurodegenerative diseases, lactylation has been recognized as a crucial modulator of macrophage function and neuroinflammation. Conditions like AD and Parkinson’s disease (PD) are distinguished by chronic inflammation and the accumulation of misfolded proteins. Recent research has underscored the significance of lactate metabolism and lactylation within the central nervous system, particularly in conditions such as AD and PD. The astrocyte-neuron lactate shuttle underscores lactate’s pivotal role in brain function, where it serves not only as an energy substrate but also as a modulator of neuronal activity and survival [113,114]. In AD, elevated levels of lactate and increased histone lactylation have been observed in senescent microglia and hippocampal tissues, suggesting a link between metabolic dysregulation and neuroinflammation. Specifically, H3K18 lactylation has been shown to activate the NF-κB signaling pathway, thereby upregulating pro-inflammatory cytokines such as IL-6 and IL-8, which contribute to the senescence-associated secretory phenotype and exacerbate neurodegeneration [99]. The levels of H4K12la are found to be increased in microglia that are located adjacent to Aβ plaques. This specific histone modification, which is dependent on lactate, shows a higher concentration at the promoters of genes involved in glycolysis, leading to the activation of transcription and a subsequent rise in glycolytic activity. Ultimately, this positive feedback loop involving glycolysis, H4K12la, and PKM2 contributes to the worsening of microglial dysfunction in AD [80]. Lactate produced during exercise promotes the phenotypic transformation of microglia, which is crucial for diminishing neuroinflammation and enhancing cognitive performance [100]. Lactate, produced by astrocytes and neurons, can induce histone lactylation in microglia, thereby influencing their activity. Transcription factor Bach1 is essential for microglia metabolic homeostasis and histone lactylation. Decreased H4K12la levels in microglia reduce histone lactylation enrichment at the promoter of Lrrc15. Microglia-derived LRRC15 participates in astrogenesis through the JAK/STAT pathway [101].

Moreover, the lactylation of YY1 facilitated the activation of microglia through the modulation of transcription for various inflammatory genes, such as STAT3, CCL5, IRF1, IDO1, and SEMA4D. This PTM of YY1 contributed to the impairment of microglial function in the context of autoimmune uveitis by enhancing the secretion of inflammatory cytokines and increasing both cell migration and proliferation [102]. The lysine-lactylation modification at p53 plays a significant role in the pro-inflammatory activation of BV2 cells induced by lipopolysaccharides under hypoxic conditions, primarily through the NF-κB signaling pathway. Furthermore, the suppression of lactate production may serve to mitigate neuroinflammatory damage [103].

Understanding the role of lactylation within the context of neurodegenerative disorders could lead to the development of innovative treatment approaches aimed at modulating this epigenetic modification to control neuroinflammation and promote neuronal survival.

### Intestinal disease

In the context of intestinal diseases, lactylation has emerged as a significant factor influencing macrophage function and the overall inflammatory response. Inflammatory bowel disease (IBD), which encompasses Crohn’s disease and ulcerative colitis, is defined by persistent inflammation within the gastrointestinal tract. Lactate produced by gut microbiota and inflamed tissues can induce histone lactylation in macrophages, thereby modulating their activity. Additionally, lactylation has been shown to influence the shift of macrophages toward an anti-inflammatory phenotype, which is crucial for maintaining intestinal homeostasis and preventing excessive inflammation. For instance, a study demonstrated that the administration of lactic acid-producing probiotics could attenuate ulcerative colitis by inhibiting macrophage pyroptosis and altering the composition of gut microbiota, highlighting the therapeutic potential of targeting lactylation pathways [105].

In addition, in colon cancer, lactate inhibits RARγ gene transcription in macrophages, leading to the activation of TRAF6-IL-6-STAT3 signaling pathway and promoting colorectal tumorigenesis [106]. Furthermore, proprotein convertase subtilisin/kexin type 9 (PCSK9) promotes the progression and dissemination of colon cancer cells through its modulation of epithelial-mesenchymal transition and the PI3K/AKT signaling pathway in tumor cells, as well as influencing the phenotypic polarization of macrophages. Focusing on the modulation of PCSK9 expression or its functional activity could be a potential therapeutic strategy for controlling colon cancer [104]. The modulation of histone lactylation in intestinal macrophages represents a promising therapeutic target for managing IBD, as it can potentially reduce inflammation and promote mucosal healing.

### Hepatic disease

Lactylation has emerged in various hepatic diseases, particularly NAFLD. The liver’s metabolic environment, rich in lactate, promotes histone lactylation, which influences gene expression in hepatic macrophages (Kupffer cells). Recent studies have highlighted the role of MPC1 in regulating FASN lactylation, which is crucial for lipid metabolism in the liver. Specifically, MPC1 expression is positively correlated with hepatic lipid deposition in NAFLD patients. In MPC1 knockout mice, a reduction in hepatic lipid accumulation was observed, which was attributed to the lactylation of FASN at the K673 site. This modification inhibits FASN activity, thereby reducing lipid synthesis and accumulation in the liver. Additionally, lactylation appears to modulate inflammation levels by influencing macrophage polarization and mitochondrial protection, suggesting a multifaceted role in NAFLD progression and treatment [82]. This modification has been linked to the regulation of genes involved in inflammation and fibrosis. In liver diseases such as NAFLD, lactylation has been identified as a key regulator of metabolic and inflammatory pathways in macrophages. The lactylation of FASN, mediated by MPC1, has been shown to inhibit its activity, thereby reducing hepatic lipid accumulation and inflammation. Additionally, lactylation of specific proteins such as FASN has been implicated in the regulation of lipid metabolism and macrophage polarization. In NAFLD, lactylation of FASN at lysine 673 inhibits its activity, leading to reduced lipid accumulation and promoting the M2 phenotype [82]. Subsequently, a comparable mechanism was observed in a model of hepatic ischemia-reperfusion injury, revealing that heat shock protein A12A may confer hepatoprotection by obstructing the activation of M1 macrophages. This protective effect is mediated through the inhibition of lactylation and the exosomal release of high-mobility group box 1 (HMGB1) [107].

Targeting lactylation pathways in hepatic macrophages could offer new therapeutic strategies for treating liver diseases by modulating inflammation and fibrosis. Additionally, interventions that reduce lactate production or inhibit histone lactylation could potentially ameliorate liver injury and improve liver function in patients with chronic liver diseases.

### Hematological disease

Lactylation has been identified in the context of sepsis, a critical medical condition marked by an abnormal immune reaction to infectious agents. Recent research indicates that lactate facilitates the lactylation process of HMGB1 within macrophages, which in turn enhances its acetylation and exosomal release, contributing to the inflammatory response in polymicrobial sepsis [75]. Furthermore, the introduction of exogenous lactate exerts harmful effects on patients suffering from sepsis. This occurs through the modulation of lactylation and acetylation modifications of HMGB1 within macrophages, which subsequently triggers the exosomal secretion of lactylated and acetylated HMGB1 from the cells. This modification is crucial as it links metabolic changes to epigenetic regulation, thereby influencing the macrophage’s role in sepsis. The interplay between lactylation and macrophage activation underscores the importance of metabolic reprogramming in the pathophysiology of sepsis.

In summary, lactylation links metabolic changes to epigenetic regulation in macrophages, influencing their roles in various human diseases. The therapeutic potential of targeting lactylation pathways offers promising avenues for the treatment of sepsis, MI, NAFLD, cancer, inflammatory diseases, neuroinflammation, and autoimmune diseases. The modulation of lactylation pathways shows potential for the advancement of targeted therapies aimed at mitigating inflammation and promoting tissue repair in various pathological conditions.

## The clinical implications of macrophage lactylation

The modulation of lactylation presents a promising diagnostic and therapeutic strategy in human diseases for future clinical implications. Diagnostic and therapeutic targets associated with lactylation in macrophages can be categorized into two types: the lactylation modification itself or the lactylation-regulatory enzymes. While the identification and analysis of lactylation proteins are still developing due to the limitations of current methods, new techniques are being created to address the complexity of differentially diagnosing protein lactylation sites. In contrast, the enzymes that regulate lactylation have been comparatively more abundantly studied and can be categorized into three groups: writers, erasers, and readers [56]. These enzymes modify protein properties through lactylation, impacting key cellular processes like stability, localization, enzyme and transcriptional activity, and interactions, thus regulating physiological and pathological processes. Understanding lactylation-regulatory enzymes is crucial for grasping lactylation’s role in human diseases.

**Figure 3:**
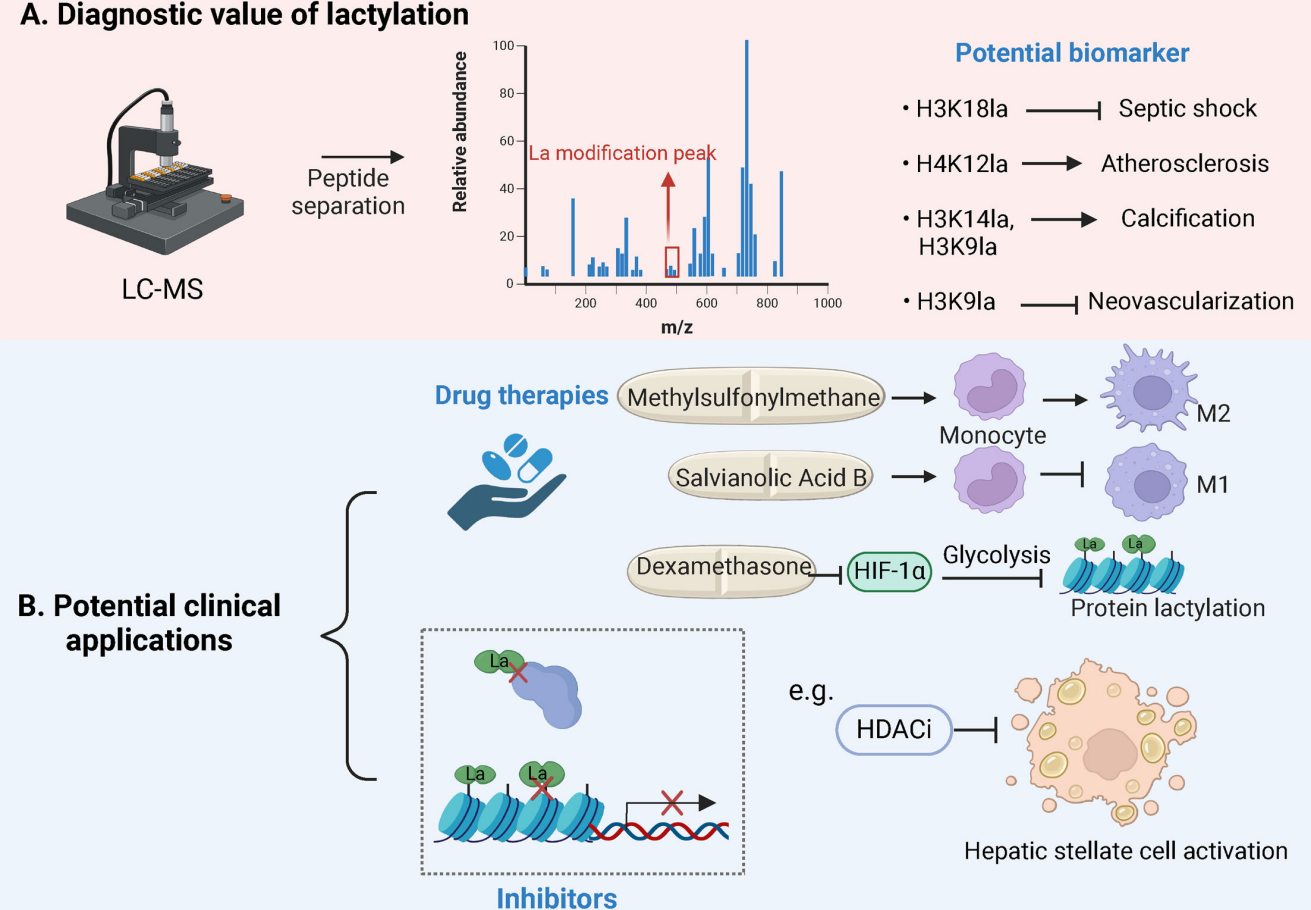
Diagnostic and potential clinical applications associated with lactylation in macrophages. (**A)** Diagnostic value of lactylation. Lactylation sites are potential diagnostic biomarkers detected by liquid chromatography-mass spectrometry (LC-MS). (**B**) Potential clinical applications. The potential therapeutic value of medicines and inhibitors may be a potential clinical treatment for lactylation acidification disease in vivo (created with BioRender.com).

Lactylation offers significant diagnostic value by correlating with disease severity in conditions as a potential biomarker for clinical assessment (Figure 3A) [115,116]. For example, H3K18la was more abundant in septic shock [109], and H4K12la offered a new therapy for vascular smooth muscle cell senescence and atherosclerosis [117]. H3K14la and H3K9la facilitate calcification and present targets for calcific aortic valve disease treatment [118]. H3K9la serves targets and disrupts pathological neovascularization [119]. By studying the correlation between specific proteins’ lactylation sites and specific diseases, new biomarkers may be identified that can indicate the state of the disease. These biomarkers can be used for early diagnosis, monitoring response to therapy, and assessing prognosis.

Lactylation acidification may be associated with disease and, therefore, has important potential clinical therapeutic value, where especially pharmacological strategies have great potential for the applications of clinical disease treatments (Figure 3B). For example, methylsulfonylmethane protects against methicillin-resistant staphylococcus aureus by promoting M2 macrophage polarization via lactate-mediated histone lactylation, offering a potential treatment for drug-resistant infections [108]. Salvianolic acid B alleviates liver injury by down-regulating glycolysis and inhibiting M1 macrophage polarization through lactate-mediated histone lactylation, suggesting its use for liver fibrosis [34]. Dexamethasone provides protective effects against asthma through the modulation of the HIF-1α-glycolysis-lactate pathway and the process of protein lactylation. Copanlisib, a PI3K inhibitor, enhances immunotherapy efficacy by counteracting macrophage-induced immunosuppression and controlling tumor growth [87,120,121]. Oxamate improves CAR-T therapy for glioblastoma by inhibiting lactylation and ectonucleotidases, which reduces lactate production and lowers H3K18 lactylation on genes associated with immunosuppression [122].

In addition to the conventional pharmacological treatments mentioned above, histone deacetylases inhibitors (HDACi) may have great therapeutic promise in terms of lactylation modification [123,124]. A novel study has found histone acetylation competes with histone lactylation, and the class I HDACi impede hepatic stellate cell activation [123]. Studies of HDACi application to the treatment of other human diseases by reducing lactylation are rare, and it provides a valuable direction for drug therapy in lactylation modification. In addition to single-drug strategies, the combination-drug strategies, combining two or more drugs, present a promising option.

Targeting lactylation pathways could offer new therapeutic strategies for disease treatment by modulating the lactylation-regulatory enzymes or the lactylation modification itself. Interventions that reduce lactate production and inhibit lactylation-regulatory enzymes or the lactylation modification itself could potentially suppress the incidence of disease and improve the efficacy of disease therapies. These therapeutic approaches underscore the potential of lactylation as a modifiable target in the treatment of inflammatory and metabolic diseases.

## Conclusions and future perspectives

The burgeoning field of lactylation, particularly in the context of macrophages, has unveiled significant insights into its multifaceted roles in human health and diseases. Balancing the diverse findings from these studies, it becomes evident that lactylation serves as a double-edged sword, with both beneficial and detrimental effects depending on lactate levels. This review highlights the molecular connections between macrophage and lactylation, as well as their regulatory mechanisms in various disease pathogenesis contexts. Furthermore, it underscores the potential clinical applications of lactylation on macrophages and opens new avenues for developing targeted interventions.

Recently, advances in the functions and mechanisms of lactylation in macrophages hold great promise for diagnostic and therapeutic applications for human diseases. However, the precise molecular mechanisms by which lactylation influences macrophage function and its broader implications in human diseases remain to be investigated in the future. First, high-throughput techniques such as mass spectrometry-based proteomics and single-cell RNA sequencing will be instrumental in mapping the lactylome and understanding its role in disease pathogenesis [125,126]. Second, understanding the cross-talk between lactylation and other PTMs will also be crucial in unraveling the complex regulatory networks that govern macrophage biology and disease pathogenesis [127-131]. These modifications cross-talk can influence protein structure and function, enhancing our understanding of biological regulation and identifying new therapeutic targets. Third, lactylation and de-lactylation are dynamic processes that are involved in regulating various cellular functions and signaling pathways. Elucidating the precise mechanisms by which lactate concentrations in different individuals influence lactylation, macrophage function, and disease outcomes can contribute to personalized medicine [132]. Additionally, the development of specific inhibitors or enhancers of lactylation could pave the way for targeted therapies that modulate macrophage function to treat inflammatory and metabolic diseases [84].

In summary, lactylation represents a critical nexus affecting various aspects of macrophage biology, particularly in their roles in immune response, inflammation, and metabolism. This modification can influence the expression of various genes involved in pro-inflammatory cytokine production and alter signaling pathways that govern macrophage activation and polarization. The dysregulation of lactylation has been linked to various human diseases, including autoimmune disorders, metabolic syndrome, and cancer, highlighting its profound implications for disease pathogenesis and progression. The ability to manipulate this modification holds promise for innovative therapeutic strategies, marking a significant step forward in medicine.

## Data Availability

The submitted article is a review and does not have any associated data files. Therefore data sharing is not applicable in this context.

## Open Access

This article has been published open access under our Subscribe to Open programme, made possible through the support of our subscribing institutions, learn more here: https://portlandpress.com/pages/open_access_options_and_prices#conditional

## Abbreviations

AD: Alzheimer’s disease
Arg1: arginase-1
Aβ: amyloid-beta
BCAP: B-cell adapter for PI3K
CXCL1: C-X-C motif chemokine ligand 1
DCM: diabetic cardiomyopathy
EAU: experimental autoimmune uveitis
EPB41L4A-AS1: erythrocyte membrane protein band 41-like 4A antisense RNA 1
FASN: fatty acid synthase
FGF2: fibroblast growth factor 2
FOXP3: forkhead box protein P3
GLUT1: glucose transporter type 1
GNAT 13: guanine nucleotide-binding protein alpha-13
GPR81: G-protein-coupled receptor 81
HACE: high-altitude cerebral edema
HK3: hexokinase-3
HMGB1: high mobility group box 1
KAT8: lysine acetyltransferase 8
KAT2B: Lysine Acetyltransferase 2B
Kla: lysine lactylation
LDHA: lactate dehydrogenase A
LTBP1: latent-transforming growth factor beta-binding protein 1
Lrrc15: leucine-rich repeat containing 15
MCT4: monocarboxylic acid transporter proteins 4
MI: myocardial infarction
MPC1: mitochondrial pyruvate carrier 1
MRE11: a crucial homologous recombination (HR) protein
NALFD: non-alcoholic fatty liver disease
NEDD4: neuronally expressed developmentally downregulated 4
PCAF: p300/CBP-associated factor P300, a multi-substrate histone acetyltransferase
PCSK9: proprotein convertase subtilisin/kexin type 9
PKM2: pyruvate kinase M2
PTMs: post-translational modifications
RARγ: retinoic acid receptor gamma
SIRT1: sirtuin 1
SLC2A1: solute carrier family 2 member 1
STAT3: signal transducer and activator of transcription 3
Sox10: Sry-related HMg-Box gene 10
TAMs: tumor-associated macrophages
VCAM1: vascular cell adhesion molecule 1
VEGF-a: vascular endothelial growth factor-a
YY1: Yin Yang 1
iNOS: inducible nitric oxide synthase
